# A Theoretical Model of Jigsaw-Puzzle Pattern Formation by Plant Leaf Epidermal Cells

**DOI:** 10.1371/journal.pcbi.1004833

**Published:** 2016-04-07

**Authors:** Takumi Higaki, Natsumaro Kutsuna, Kae Akita, Hisako Takigawa-Imamura, Kenji Yoshimura, Takashi Miura

**Affiliations:** 1 Department of Integrated Biosciences, Graduate School of Frontier Sciences, The University of Tokyo, Tokyo, Japan; 2 Research and Development Division, LPixel Inc., Tokyo, Japan; 3 Department of Anatomy and Cell Biology, Graduate School of Medical Sciences, Kyushu University, Fukuoka, Japan; 4 Department of Neurology, Osaka City General Hospital, Osaka, Japan; Duke University, UNITED STATES

## Abstract

Plant leaf epidermal cells exhibit a jigsaw puzzle–like pattern that is generated by interdigitation of the cell wall during leaf development. The contribution of two ROP GTPases, ROP2 and ROP6, to the cytoskeletal dynamics that regulate epidermal cell wall interdigitation has already been examined; however, how interactions between these molecules result in pattern formation remains to be elucidated. Here, we propose a simple interface equation model that incorporates both the cell wall remodeling activity of ROP GTPases and the diffusible signaling molecules by which they are regulated. This model successfully reproduces pattern formation observed *in vivo*, and explains the counterintuitive experimental results of decreased cellulose production and increased thickness. Our model also reproduces the dynamics of three-way cell wall junctions. Therefore, this model provides a possible mechanism for cell wall interdigitation formation *in vivo*.

## Author Summary

It is well known that plant epidermal cells show beautiful jigsaw-puzzle like pattern. However, mechanism of this pattern formation is not well understood. In this study, we integrated known experimental information and mathematical modeling to reproduce the main features of the pattern formation—maintenance of cell wall thickness and formation of interdigitation. Interestingly, the model is mathematically equivalent to the model of human skull suture interdigitation.

## Introduction

**Fig 1:**
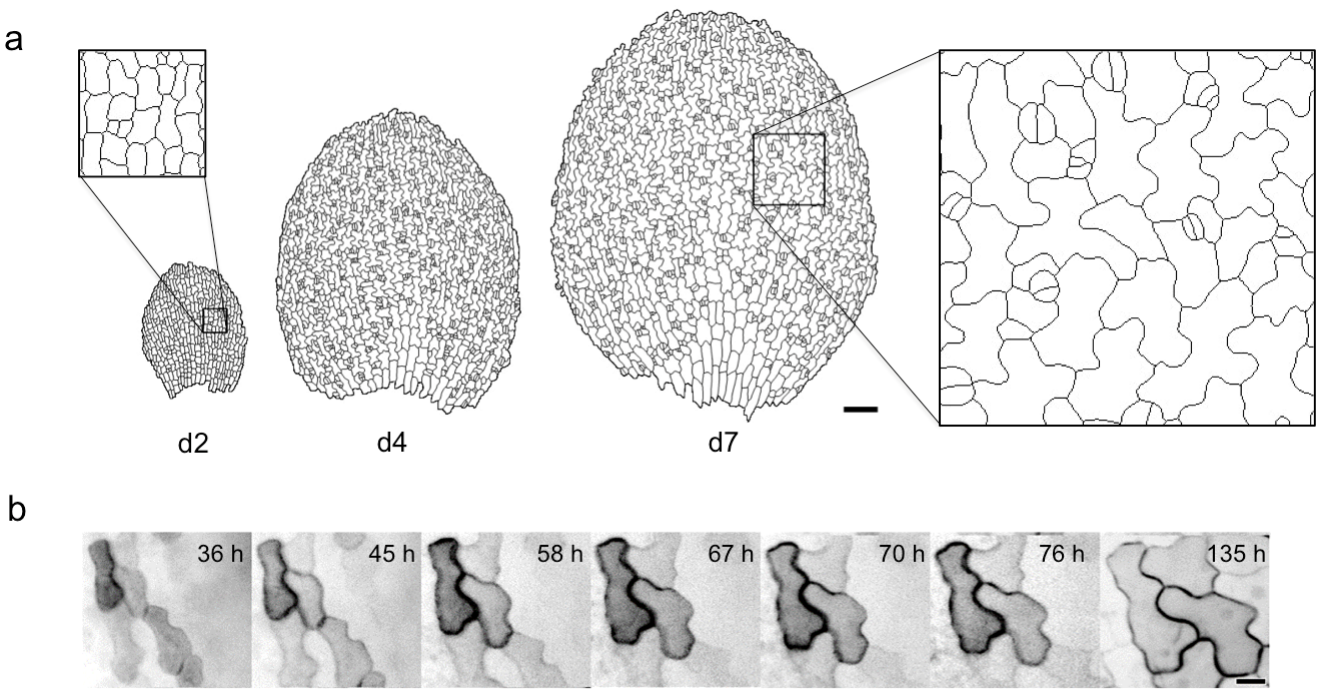
Jigsaw-puzzle pattern formation by leaf epidermal cells in *Arabidopsis thaliana*. (a) Traced image of epidermal cell wall in an abaxial cotyledon surface. Cell contours were visualized with the plasma membrane marker GFP-PIP2a on the cotyledon surface of seedlings 2 days (left), 4 days (middle) and 7 days (right) after sowing. Scale bar = 100 μm. (b) Time sequential images of abaxial cotyledon epidermal cells. Scale bar = 10 μm.

Throughout growth and differentiation, plant cells display various shapes that are primarily determined by the cell wall [1,2]. Leaf epidermal cells in dicotyledonous plants have jigsaw puzzle–like shapes with winding cell wall [3,4]. Prior to leaf expansion, epidermal cells have a simple rectangular shape. The cell wall begins to wind during leaf expansion, forming interdigitated cell patterns [5–7]. In the cotyledons of *Arabidopsis thaliana*, significant winding is observed for approximately one week after seed sowing (Fig 1). Both the cell volume and total cell wall length of an epidermal cell increase as it changes in shape. During this process, the thickness of the cell wall remains mostly unchanged, but traditional transmission electron microscopic observations suggest that the cell wall becomes slightly thicker as an accompaniment to cortical microtubule accumulation in the winding zone [8].

Cell wall interdigitation is regulated by two Rho-like GTPases from plants (ROPs), ROP2 and ROP6 [5,9,10]. ROP2 and ROP6 have opposing activities; the activity of ROP2 dominates under low auxin concentrations, whereas ROP6 activity becomes dominant under high auxin concentrations [10]. ROP2 localizes to protrusions of epidermal cells and promotes localization of diffuse F-actin, which enhances outgrowth via targeted exocytosis [5,11] as observed in tip growth [12]. In contrast, ROP6 localizes to the concave region and accumulates cortical microtubules [13] that likely restrict cell expansion via cell wall reinforcement [8,14].

The general mechanisms underlying the formation of similar biological patterns have been examined using a reaction–diffusion framework. Pattern formation by the reaction–diffusion system has been widely investigated in the field of mathematical biology [15], and the findings obtained were recently used by developmental biologists [16]. The dynamics of winding of a band-like structure, which are similar to the dynamics of interdigitation of the plant cell wall, have been modeled using the FitzHugh–Nagumo equation [17,18]. The dynamics of winding are controlled by the combination of two mechanisms: maintenance of the band-like shape by the interaction of two interfaces and formation of curvature due to interface instability. This mechanism has been applied to the interdigitation of the junctions between bones in human skulls [19].

In the present study, we formulate a theoretical model to reproduce pattern formation by plant leaf epidermal cells. This model assumes that the interdigitating pattern arises as a result of cell wall remodeling, and reproduces the maintenance of cell wall thickness and formation of a jigsaw puzzle–like pattern *in vivo*.

## Materials and Methods

### Time-lapse observations

To monitor epidermal cell morphogenesis, time-lapse imaging of the cotyledon surface was performed with *A*. *thaliana* seedlings as described previously [20]. Sterilized seeds expressing the plasma membrane marker GFP-PIP2a [21] were immersed in distilled water at 4°C for 2 days, and the seed coats were then carefully removed under a stereo microscope (SZX12, Olympus, Tokyo, Japan). The naked cotyledons were mounted on a chamber slide (Iwaki Co., Ltd, Tokyo, Japan) and covered with 1/2-strength Murashige–Skoog medium agar gel (2.3 g L^−1^ Murashige and Skoog Plant Salt Mixture, pH 5.8 from Wako Pure Chemical Industries, Osaka, Japan). The chamber slides were placed in growth chambers at 23.5°C, with a 12-h light/12-h dark cycle, using 100 μmol m^−2^ s^−1^ white light. For acquiring images, the chamber slide was placed onto the inverted platform of a fluorescence microscope (IX70, Olympus) equipped with a UPlanFl 20×/0.50 objective lens and spinning disc confocal unit (CSU10, Yokogawa Electric Co., Ltd, Tokyo, Japan), together with a cooled CCD camera head system (CoolSNAP HQ; Photometrics, Huntington Beach, Canada).

### Cellulase treatment

Sterilized *A*. *thaliana* seeds expressing GFP-PIP2a [21] were immersed in 1/2-strength Murashige-Skoog media solution (2.3 g L^−1^ Murashige and Skoog Plant Salt Mixture, pH 5.8 from Wako Pure Chemical Industries) supplemented with or without 1.0% cellulase (Cellulase Y-C; Kyowa Chemical Products Co., Ltd, Osaka, Japan) in 24-well plates (Sumitomo Bakelite Co., Ltd, Tokyo, Japan). The seeds were cultured for one week in growth chambers at 23.5°C, with a 12-h light/12-h dark cycle using 100 μmol m^−2^ s^−1^ white light, and then observed with a confocal laser scanning microscope (FV300, Olympus).

### Transmission electron microscopy

To observe the cell wall ultrastructure, we observed the lateral cell wall of cotyledon epidermal cells with transmission electron microscopy. Cotyledon samples were fixed with 2% paraformaldehyde and 2% glutaraldehyde in 0.05 M cacodylate buffer (pH 7.4) at 4°C overnight. After fixation, the samples were rinsed three times with 0.05 M cacodylate buffer for 30 min each, followed by post fixation with 2% osmium tetroxide in 0.05 M cacodylate buffer at 4°C for 3 hours. The samples were dehydrated through a graded ethanol series (50% ethanol for 30 min at 4°C, 70% ethanol for 30 min at 4°C, 90% for 30 min at room temperature, and 4 changes of 100% for 30 min each at room temperature). Afterwards, the samples were continuously dehydrated with 100% ethanol at room temperature overnight. The samples were infiltrated with propylene oxide twice for 30 min each and then placed into a 70:30 mixture of propylene oxide and resin (Quetol-651; Nisshin EM Co., Tokyo, Japan) for 1 hour. The cap of the tube was left open and propylene oxide was evaporated overnight. The samples were transferred to fresh 100% resin, and polymerized at 60°C for 48 hours. 80 nm sections were sliced from the blocks using an ultramicrotome equipped with a diamond knife (ULTRACUT UCT; Leica, Tokyo, Japan), and sections were placed on copper grids. They were stained with 2% uranyl acetate at room temperature for 15 minutes, rinsed with distilled water, and counter-stained with lead stain solution (Sigma-Aldrich Co., Tokyo, Japan) at room temperature for 3 minutes. The grids were observed under a transmission electron microscope (JEM-1400Plus; JEOL, Ltd., Tokyo, Japan) at an acceleration voltage of 80 kV. Images were taken with a CCD camera (VELETA; Olympus). Lateral cell wall thickness was measured at the thinnest point between two three-way junctions to avoid errors due to the direction of the cuts.

### Image quantification for the wavenumber of the cell wall and angles at three-way junctions in leaves

We obtained cell contour images of GFP-PIP2a-expressing plants [21] or *rsw2/kor1* mutant lines [22,23] stained with the fluorescent dye FM4-64. The images obtained were thresholded by pixel intensity and skeletonized to segment the cell wall pattern. As inhomogeneous fluorescence signal was occasionally observed, we manually corrected defects in the cell wall pattern in the segmented images. We then extracted all cells in the upper leaf regions and measured the cell area. To quantify the ratio of the wavenumber of the cell wall, we used a G-type Fourier descriptor, which generates a power spectrum from a closed curved shape, such as a two-dimensional representation of a leaf (S1 Fig).

The angles of three-way junctions in each cell were measured at points that were 12 pixels from the central pixel of the junction, as shown in S2 Fig. Deviation of the angle from 120° was evaluated by the root-mean-square deviation (RMSD) of each cell as follows:

$$\text{RMSD}=\;\sqrt{\frac{1}{N}\Sigma_{i=1}^{N}{\left(\theta_{i}-120^{\text{o}}\right)}^{2}}$$ (1)

where *θi* is the angle of the *i*-th three-way junction in the cell and *N* is the number of junctions in the cell.

### Quantification of microtubule distribution

To evaluate the density of anticlinal cortical microtubules in epidermal cells, we observed the cotyledon surfaces of transgenic *A*. *thaliana* plants expressing GFP-tubulin [24,25] 8 days after sowing in 1/2-strength Murashige-Skoog solution. The three-way junctions and points of interdigitation were manually determined from GFP-tubulin maximum intensity projection images obtained from serial optical sections at 0.5-μm steps along Z-axis. Following this, GFP intensity peaks were semi-automatically detected as anticlinal microtubules within circles centered at the manually assigned points with a radius of 5 μm. The density of the anticlinal microtubules was calculated as the number of GFP intensity peaks per cell surface length.

### Model

**Fig 2:**
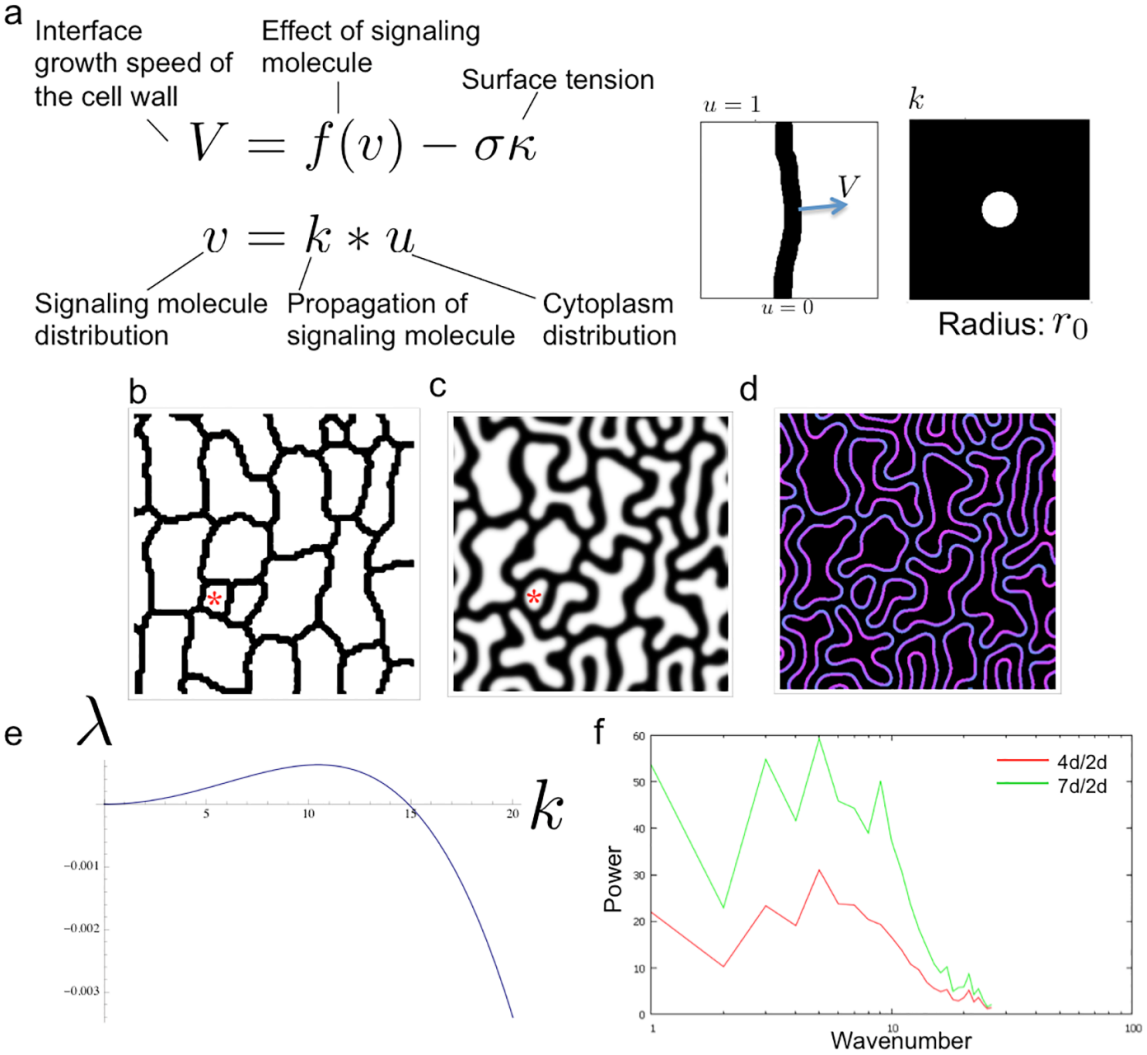
Description of the model. (a) Governing equation of the model. (b) Initial distribution of cell wall taken from measurements of seedlings. Black indicates the cell wall; white indicates the cell interiors. Red asterisk indicates a small stomatal lineage cell. (c) Cell wall interdigitation at *t* = 100. Red asterisk indicates a small stomatal lineage cell. (d) Signaling molecule concentration at the interface. The protruded side had a higher ROP2/ROP6 ratio. Cyan indicates ROP2 activity; magenta indicates ROP6 activity. (e) Dispersion relation of the model obtained by a linear stability analysis. The specific wavenumber component grew. (f) The ratio of wavenumber components obtained by analysis of *in vivo* cell wall pattern changes. The upper leaf regions of 2-, 4-, and 7-day-old seedlings were analyzed. The red line indicates the ratio between 4- and 2-day-old seedlings in each wavenumber. The green line indicates the ratio between 7- and 2-day-old seedlings in each wavenumber.

We formulated a model that incorporated local remodeling of the cell wall in an attempt to understand pattern formation within the cell wall during interdigitation (Fig 2a). Our model includes the following assumptions:

1. The cell wall is remodeled at the interface between the cell wall and plasma membrane.
2. Remodeling of the cell wall is regulated by local concentrations of active ROP proteins.
3. ROP protein activity is determined by the concentration of a small signaling molecule.
4. The signaling molecule is produced in the cytoplasm, transported into the cell wall, and exerts its influence up to a certain distance.

We initially defined the indicator variable *u*(*x*,*y*), which represents the structure at a certain location (*x*,*y*). We defined the *u =* 1 region as the cytoplasm and the *u =* 0 region as the cell wall. *v* represented the local signaling molecule concentration. We then defined the interface speed *V* as follows:

V = f(v) − σκ (2)

This equation means that local remodeling of the cell wall is a function of the local signaling molecule concentration and curvature of the cell wall. We represented the effects of the signaling molecule as *f*(*v*), where *v*(*x*,*y*) is the spatial distribution of signaling molecule. At interface points where the local signaling molecule concentration is high, ROP6 becomes active and the cell wall is degraded, resulting in *V* < 0. Conversely, if the local concentration of signaling molecule is low, ROP2 becomes dominant and cell wall is produced, resulting in *V* > 0. As the cell wall is elastic, we also introduced the surface tension term *σk*, which inhibits the formation of pointy structures at the interface. This type of interface equation can be calculated using the phase field method.

We then used a convolution kernel to implement the effects of signaling molecule on cell wall remodeling. Cell wall interdigitation is relatively slow compared to the diffusion and degradation of typical signaling molecules, taking approximately one week to produce the final jigsaw puzzle–like pattern (Fig 1). Therefore, we assumed that the distribution of signaling molecule was in a quasi-steady state and calculated its distribution separately. We can calculate the distribution of signaling molecule by solving the diffusion equation; however, to simplify the model, we used a convolution kernel. We defined the convolution kernel *k*, which represents the effects of a small piece of the cytoplasm on the distribution of signaling molecule. The distribution *v*(*x*,*y*) was calculated by

v = k ⊗ u (3)

where ⊗ represents convolution. For simplicity, we used the kernel shape

$$k=1/\left(\pi r^{2}\right)\;\left(x^{2}+y^{2}<r_{0}^{2}\right)$$ (4)

$$k=0\;\left(x^{2}+y^{2}\geq r_{0}^{2}\right)$$ (5)

where *r*_0_ represents the effective range of signaling molecule influence.

### Numerical simulation

The interface equation model was implemented using the phase field method [26]. In this method, the interface equation

V = f(v) – σκ (6)

was calculated by the Allen–Cahn equation

$$u\prime \;=\;a\;u\left(1-u\right)\;\left(u-1/2\;+\;b\;f\left(v\right)\right)\;+\;d_{u}\Delta u.$$ (7)

A numerical simulation was implemented in *Mathematica* (Wolfram Research Inc., Champaign, U.S.A., S1 Code.). An implicit method was used to calculate the diffusion term of the model. Convolution was calculated using Fourier transformation. The source code of all numerical simulations is available as electronic supplemental data.

## Results

### Time-lapse observations of epidermal cell pattern formation

To observe pattern formation in interdigitating cotyledon epidermal cells, we used *A*. *thaliana* plants expressing a fluorescently tagged plasma membrane protein, GFP-PIP2a. Two days after sowing, epidermal cells were rectangular (Fig 1a; day 2). As the plant developed over 2–5 days and cells grew, cell wall gradually became bent, resulting in a jigsaw puzzle–like pattern (Fig 1a; days 4, 7). Cell wall interdigitation was confirmed by time-lapse imaging (Fig 1b).

### Interface equation to reproduce pattern formation by the cell wall

We performed a numerical simulation with our model using an appropriate initial distribution of cell wall (Fig 2b). Our simulation recapitulated interdigitation (Fig 2c and 2d). Most parts of the cell wall retain a uniform thickness, but the thickness is variable in interdigitated regions (Fig 2c). Winding is not observed in the cell wall of small stomatal lineage cells (Fig 2c and 2d, asterisk). According to our model, the convex interfaces of interdigitated cells had a lower concentration of signaling molecule, while the concave interfaces had a higher concentration (Fig 2d). As the concentration of signaling molecule is expected to influence the ratio of ROP2 and ROP6 signaling activities, the observed differences in our simulation may reflect *in vivo* modulation of ROP activity.

We performed a linear stability analysis of the model. The growth speed *λ* and wavenumber *k* are represented as

$$\lambda =\frac{a}{\pi r^{2}}\left(\varsigma \left(k,\;r\right)\;+\;\phi \left(k\right)\;\text{cos}\;\psi -2\sigma \right)-bk^{2}$$ (8)

Where

$$\phi \left(k\right)\;=\;\frac{2\;\text{sin}k\;\sigma }{k}$$ (9)

and

$$\varsigma \left(k,\;\text{r}\right)\;=\;2\;r\;\left(1\;–\frac{\text{sin}\;kr}{kr}\right)$$ (10)

[27]. *ψ* represents the phase difference between two interphases, and *λ* achieves maximum when *ψ* = 0. We plotted the relationship between *λ* and *k*, which shows a vault-like shape (Fig 2e). This shape indicates that a specific wavenumber was selected at the onset of pattern formation. The maximum value of this curve corresponds to the fastest growing wavenumber of the pattern. This analysis allows us to predict the size of the structure (as determined by the fastest growing wavenumber), but phase of the pattern is dependent on initial small perturbations and thus not predictable.

To directly compare the predictions of the simulation with our experimental observations, we calculated the ratio of the power spectra at two different time points of growth. We defined the time interval between the two stages as Δ*t*, such that the ratio of power spectra was anticipated to be

$$\frac{u_{0}e^{\lambda \left(k\right)\;\left(t+\Delta t\right)}}{u_{0}e^{\lambda \left(k\right)\;t}}=\;e^{\lambda \left(k\right)\;\Delta t}$$ (11)

We calculated the G-type Fourier descriptor of each cell, and calculated the ratio between 2-day-old and 4-day-old seedlings, and 2-day-old and 7-day-old seedlings (red and green in Fig 2f, respectively). In the G-type descriptor, the spectrum at *k* = 1 represents growth of the cell and was not similar to the result described in Fig 2e. In other wavenumbers, both distributions were vault-like, indicating that the dynamics observed *in vivo* are qualitatively similar to those predicted by the model.

### Effects of cell wall degradation

**Fig 3:**
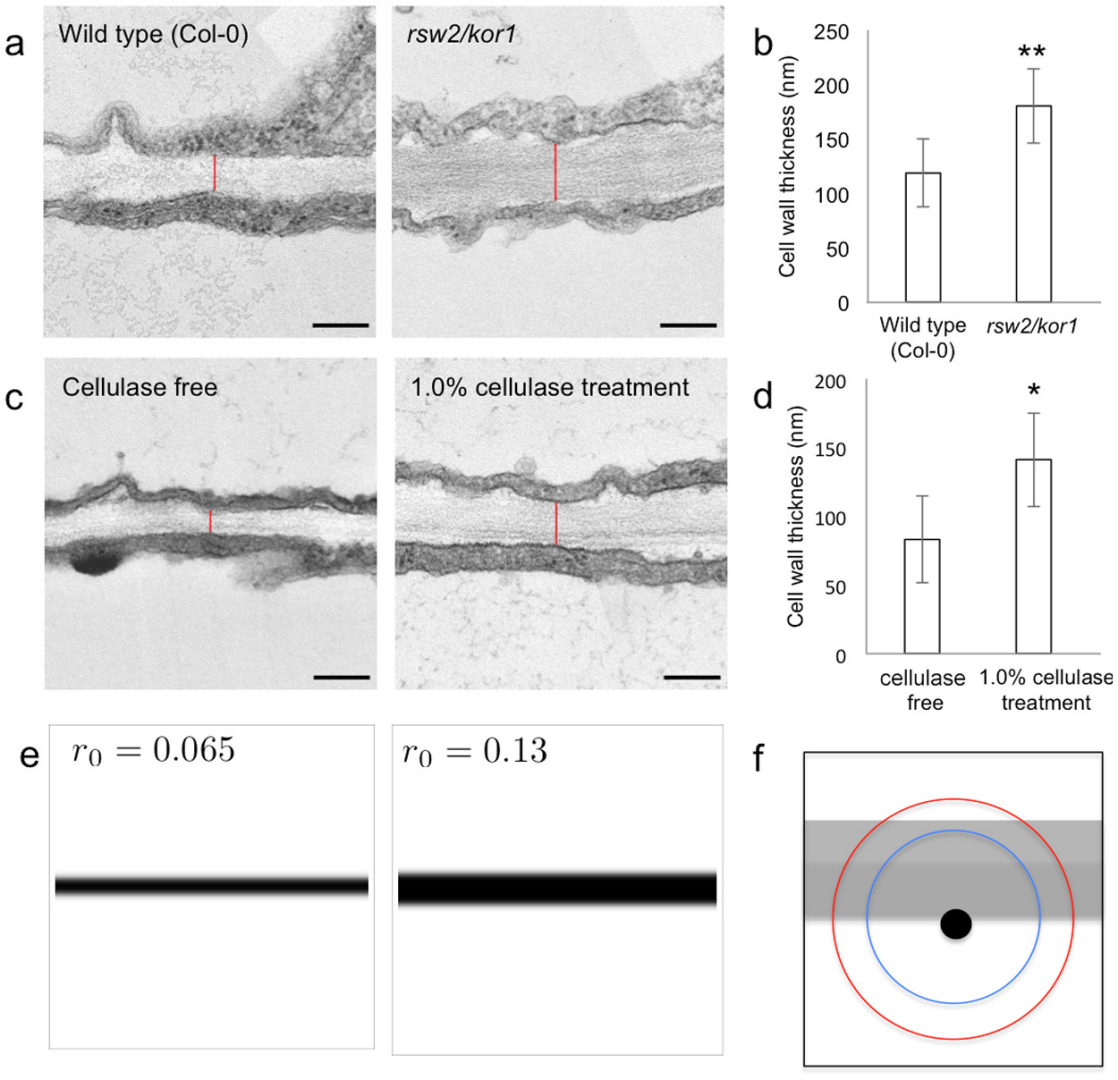
Effects of decreased cellulose on cell wall thickness. (a) Transmission electron microscopic images of the cotyledon lateral cell wall in an *rsw2/kor1* mutant, in which cellulose production is decreased. The red line shows the thickness of the cell wall. Scale bars = 200 nm. (b) The mean thickness of the cotyledon lateral cell wall in the wild type (Col-0) and *rsw2/kor1* mutant plants 7 days after sowing. Data are mean ± SD (*n* = 5). **p < 0.005 (U-test). Note that thickness increased in the *rsw2/kor1* mutant. (c) Transmission electron microscope images of the cotyledon lateral cell wall in wild-type seedlings with or without cellulose treatment. The red line shows the thickness of the cell wall. Scale bars = 200 nm. (d) The mean thickness of the cotyledon lateral cell wall after 7 days in wild type plants (Col-0) with or without cellulase treatment. Data are mean ± SD (*n* = 6). *p < 0.01 (U-test). Note that thickness was increased by cellulase treatment. (e) A numerical simulation of the model in which the effective range of signaling molecule was changed. The thickness of the cell wall increased by increasing the effective range of action of signaling molecule. (f) Model of cell wall thickness change. A certain point of the cell wall-cytoplasm interface detects the amount of cytoplasm around that point, and its detection range is defined by the kernel diameter (blue circle). The boundary reaches a steady state when the interface speed becomes zero due to the signaling molecule effect from the neighboring cell. If the kernel diameter is larger (red circle), the effect comes from further away and, the steady-state thickness increases as a result.

To examine the influence of cell wall metabolism on pattern formation, we investigated the effects of cellulose synthase dysfunction in *rsw2/kor1* mutant plants [22,23] or the enzymatic degradation of cellulose in wild-type plants treated with 1.0% cellulase for 7 days. A thicker lateral cell wall was observed in cotyledon epidermal cells from *rsw2/kor1* seedlings (Fig 3a). Cellulase treatment also resulted in a thicker cell wall (Fig 3c). We confirmed this with a statistical analysis (Fig 3b and 3d). These observations are seemingly counterintuitive but agree with previous reports about *rsw2/kor1* mutants [23]. It is previously suggested that deposition of other cell wall components such as pectin is dramatically increased to compensate for decreased cellulose [28,29].

To model the effect of impaired cellulose deposition on pattern formation, we assumed that decreased cellulose in the cell wall permits increased diffusion of signaling molecule and thus increases its range of action. By increasing the kernel radius *r*_0_, our model reproduced increased cell wall thickness (Fig 3e). An intuitive explanation of this phenomenon is as follows: the thickness of the cell wall becomes stable when *V* = *f*(*v*) = 0. *f*(*v*) approaches 0 when the effects of adjacent cells are balanced by the intrinsic effects of cell wall degradation. Therefore, if the distance at which the signaling molecule is effective increases, the cell wall becomes thicker (Fig 3f).

**Fig 4:**
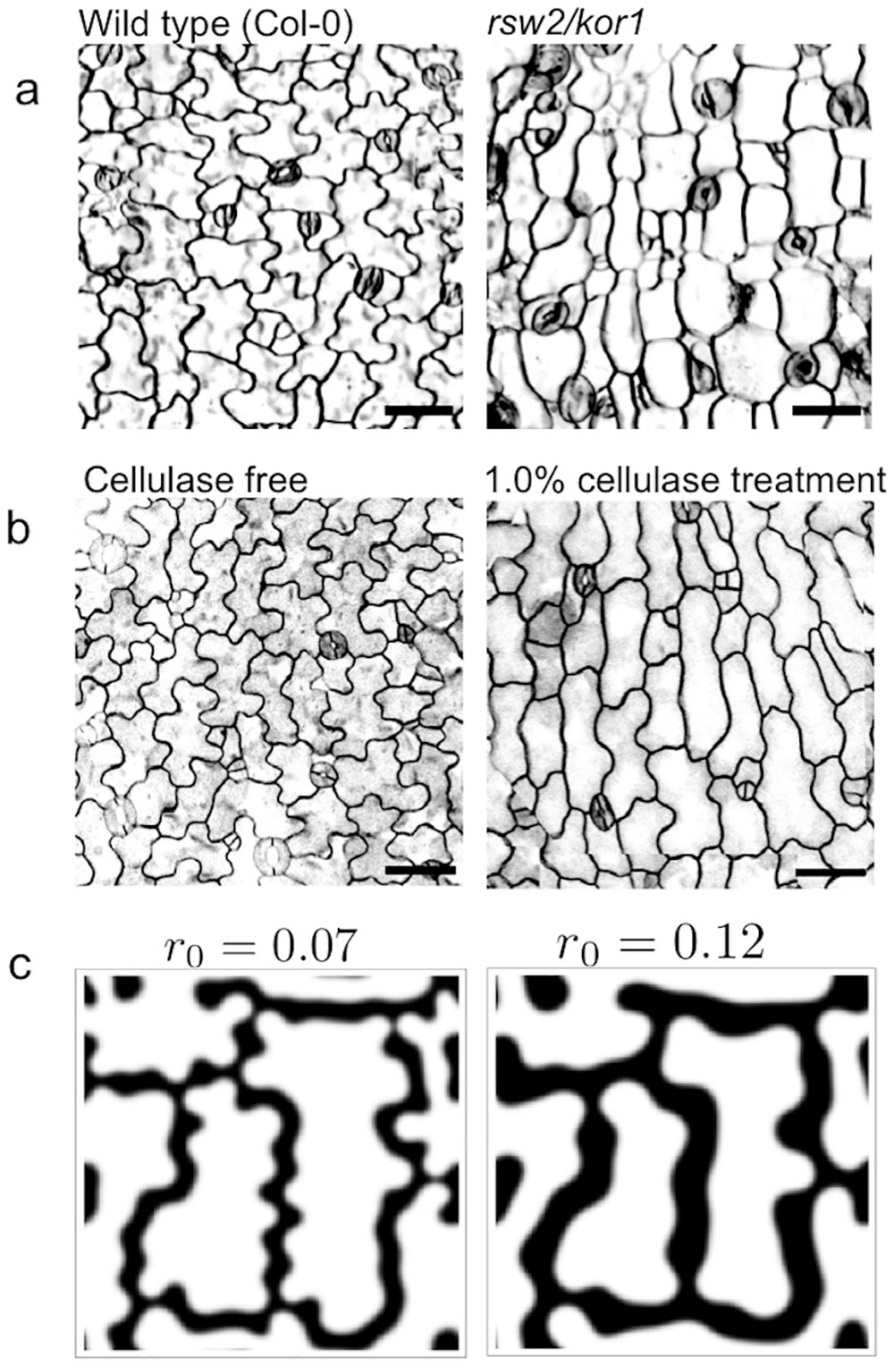
Effects of cellulose degradation on cell wall interdigitation. (a) The *rsw2/kor1* mutant displayed less curvature. Scale bars = 50 μm. (b) Effects of cellulase treatment on curvature formation. Curvature formation was also inhibited. Scale bars = 50 μm. (c) A numerical simulation of the model with a different range of action of signaling molecule. Less interdigitation occurred when the signaling molecule range of action increased, which is consistent with experimental observations.

Cell wall curvature was decreased in both *rsw2/kor1* mutants and cellulase-treated seedlings (Fig 4a and 4b). We also reproduced this decrease in cell wall curvature and increase in the characteristic length of the pattern by increasing the range of the effect *r*_0_ (Fig 4c).

### Dynamics of three-way junctions

**Fig 5:**
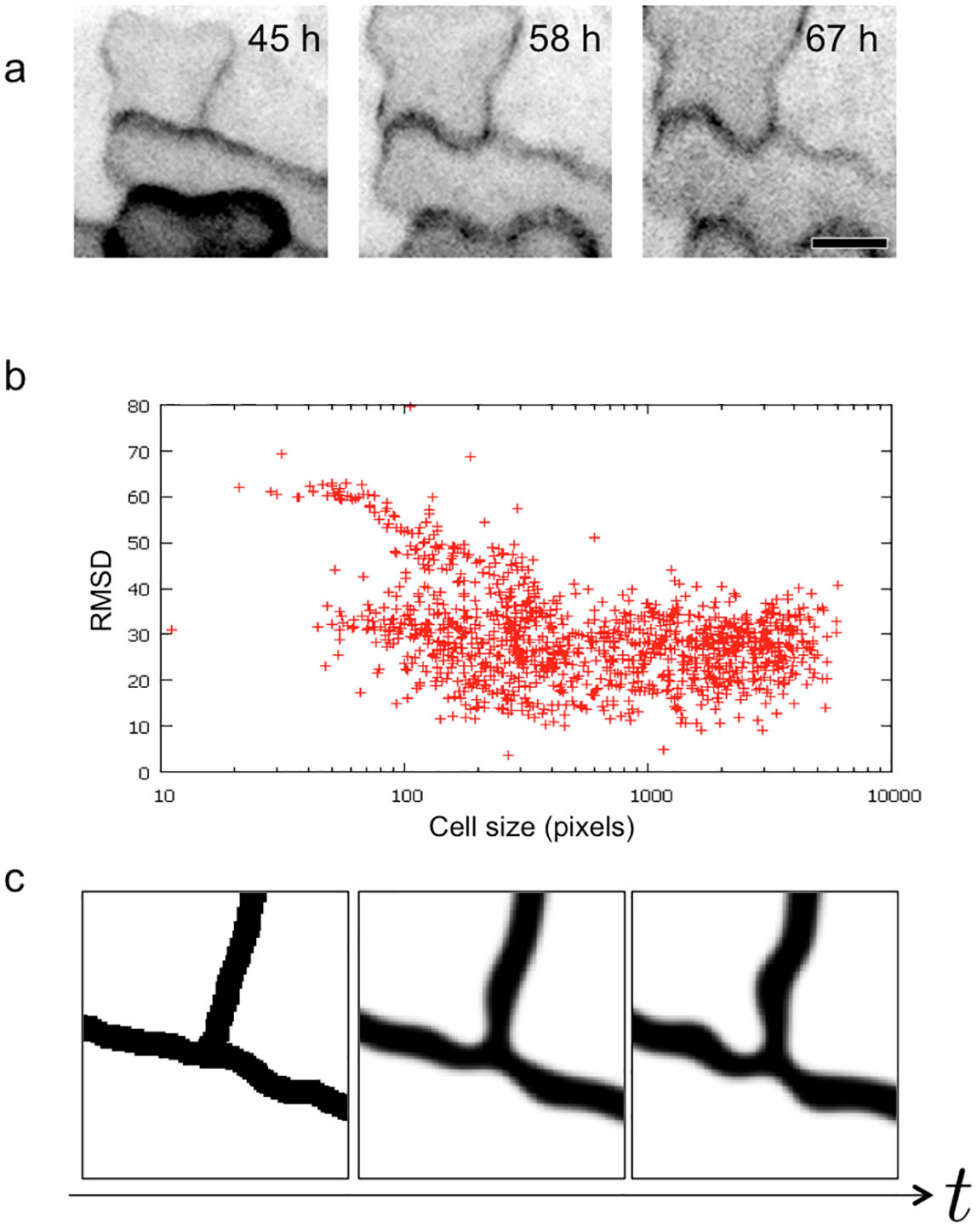
Characteristics of cell wall three-way junctions. (a) Time-lapse observation of a three-way junction using the membrane marker GFP-PIP2a. The angles were mostly 120°. Scale bar = 10 μm. (b) The relationship between cell size and the degrees of three-way junctions 7 days after sowing. Larger, more developed cells were more likely to have angles closer to 120°. RMSD, root-mean-square deviation. See also S3 Fig for data from samples 2 and 4 days after sowing. (c) Snapshots of the numerical simulation of the model at a three-way junction. The numerical simulation shows that the angles of a three-way junction gradually approach 120°.

The cell wall in young epidermis displayed a brick wall–like pattern with T-shaped three-way junctions. As development proceeded, the angles between each segment of cell wall at a three-way junction gradually approached 120° (Fig 5a). To quantify this, we automatically detected three-way junctions in cell wall and calculated the RMSD from 120° in the angles of junctions. RMSD is at a minimum when all three angles at a three-way junction are 120°. As development proceeds, cells become larger (S3 Fig). The RMSD of three-way junction angles in a population of smaller cells is biphasic, which represents three-way junctions with angles of 90°, 90°, and 180°. The RMSD of three-way junction angles is smaller in larger cells, indicating that the angles at three-way junctions all gradually approach 120° during development (Fig 5b).

We also observed this trend in our numerical simulation (Fig 5c). The angles at three-way junctions gradually became 120° over the course of the numerical simulation. This symmetry can be intuitively explained by the fact that the effects of the cytoplasm are equivalent from all three adjacent cells, and, as a result, the patterns of equal angles are expected to be the most stable.

**Fig 6:**
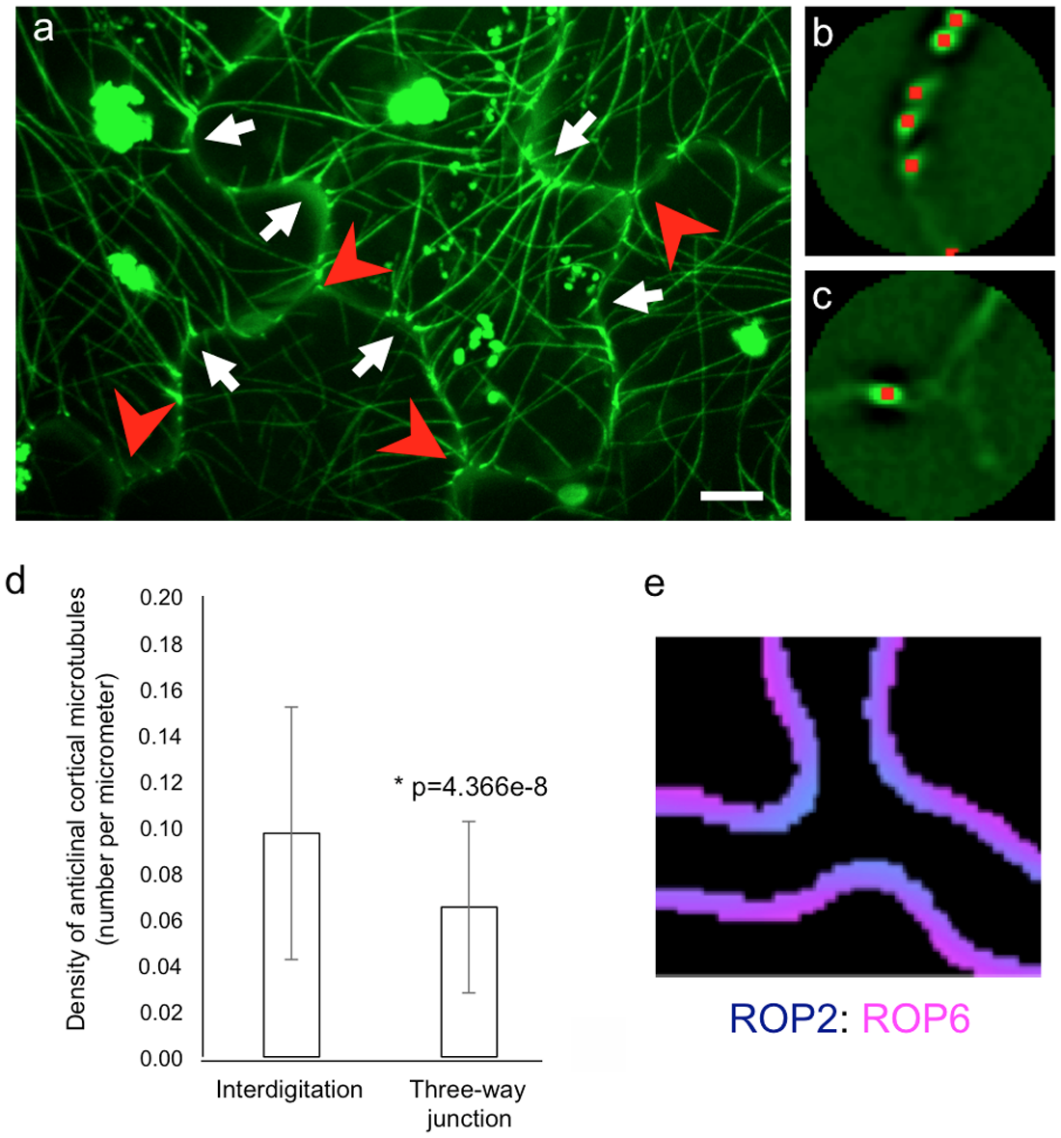
A three-way junction became a forced cytoplasmic protrusion and may affect downstream molecular pathways. (a) Maximum intensity projection of GFP-tubulin in cotyledon leaf epidermal cells. White arrows and red arrowheads indicate pavement interdigitation and a three-way junction, respectively. Scale bar = 10 μm. (b, c) A representative circle with a radius of 5 μm centered at an interdigitation point (b) and a three-way junction point. (c) Red points indicate GFP-tubulin intensity peaks detected as anticlinal cortical microtubules. (d) Density of anticlinal cortical microtubules in interdigitation and three-way junction areas. Data are mean values from 134 and 161 independent regions, respectively. A statistically significant difference is observed using the Mann–Whitney U-test (p = 4.366e−8). (e) Relative strength of ROP2 (cyan) and ROP6 (magenta) activities in a numerical simulation. The three-way junction region functioned as a forced cytoplasmic protrusion at which the ROP2 pathway was expected to be dominant. ROP2 activity was monitored by the distribution of cortical microtubules.

Based on this model, we were able to predict that three-way junctions were forced protrusions of the cytoplasm, resulting in decreased ROP6 activity in these regions (Fig 6e). To experimentally verify this prediction, we observed the distribution of cortical microtubules, which are known to be stabilized by ROP6 [10] (Fig 6a). We used transgenic *A*. *thaliana* plants expressing GFP-tubulin to observe anticlinal cortical microtubules in interdigitated cells. We semi-automatically detected GFP-tubulin signal intensity peaks and counted them in interdigitated regions (Fig 6b) and three-way junctions (Fig 6c) separately. The density of anticlinal cortical microtubules was decreased in three-way junctions (Fig 6d), consistent with the model prediction.

## Discussion

In the present study, we reproduced the pattern formation process *in vivo* using a mathematical model by incorporating known molecular interactions. Our model simultaneously recapitulated both maintenance of cell wall thickness and interdigitation of developing cells. The effects of cell wall degradation and dynamics of three-way junctions observed in our experiments were also consistent with predictions of our model.

### Intuitive explanation of pattern formation

Our model postulates that the range at which a signaling molecule can exert its effects underlies the maintenance of cell wall thickness. If a cell wall is too thick, the effects of signaling molecule on one side of the cell wall cannot reach the other side, resulting in a relatively ROP2 dominant state at that region. The wall then retracts, resulting in a thinning of the cell wall. In contrast, if the cell wall is too thin, the cell wall is strongly affected by signaling molecule produced in an adjacent cell, resulting in a ROP6 dominant state and thickening of the cell wall. Cell wall thickness is thus kept constant by the balance of these two opposed mechanisms. Therefore, the thickness of the wall is similar to the range at which signaling molecule can function. Cellulase treatment may change the range of action of signaling molecule by changing the composition and thus diffusion coefficient of cell wall. Compensatory production of other cell wall materials such as pectin may also result in thickening of cell wall, and experimental verification is necessary to distinguish these two mechanisms.

Our model also reproduces the formation of cell wall interdigitation. We considered the case in which the cell wall is slightly bent. Protruding cytoplasm near a concave region of the cell wall may be exposed to a lower concentration of signaling molecule because it is surrounded by less signaling molecule-producing cytoplasm. Therefore, ROP2 becomes dominant at that point, resulting in further lobing of the cytoplasm. In contrast, a convex area is exposed to a higher concentration of signaling molecule than a concave area because the area is surrounded by more signaling molecule-producing cytoplasm. Therefore, ROP6 becomes dominant at that point, resulting in further retraction of the cytoplasm. In a curved region of cell wall, both sides of the cell wall-cytoplasm interface tend to generate the same curvature, and as a result it, is difficult to retain the same cell wall thickness. Therefore, cell wall thickness tends to vary in the curved regions.

Because only a specific wavenumber component grew in our model, large structures cannot grow in a small domain. Stomatal guard cells are generally smaller than other epidermal cells and do not show a jigsaw puzzle–like pattern. One possibility is that stomatal guard cells have a different cell wall composition that resists curving. However, we observed jigsaw puzzle–like stomatal guard cells in a mutant line with giant stomatal guard cells [30]. Our model does not generate the pattern when the domain size was below a certain threshold. Therefore, the reason we do not observe pattern formation in wild-type stomatal guard cells is that these cells are too small to generate a pattern.

### Other possible mechanisms

A mechanical factor may be also involved in pattern formation. A previous study attempted to explain plant cell shape from a purely mechanical perspective [31]. Pattern formation of suture tissue has also been previously explained from a primarily mechanical point of view [32,33]. Our model included the surface tension term *σk* that represents the mechanical aspect of cell wall. We also postulated that pattern formation by the cell wall may be due to buckling instability (Takigawa-Imamura *et al*., in prep). It is possible to reproduce pattern formation with a mechanical model, but, in this case, we required a biological mechanism to increase the cell wall area while maintaining cell wall thickness. In cell membranes, the thickness of the membrane is automatically determined by the unit size, whereas cell wall may have variable thickness. Because cell size increases during development (Fig 5b), we cannot assume that buckling is caused by a relative decrease in cell volume.

ROP GTPases self-organize to form patterns within a cell, even without significant changes in cell shape [34]. Our model used cell geometry for interface instability, and did not include this mechanism. If we assume that intracellular processes are the primary mechanism generating cell wall patterns, we need to implement a mechanism to keep cell wall thickness constant. This mechanism and our model are not mutually exclusive. A model that includes both mechanisms has been proposed in a different context [35]. In this case, the intrinsic pattern formation mechanism modified the basic branched structure of *Drosophila* sensory neurons.

### Perspective

Our model explains interdigitation and maintenance of cell wall thickness, but it does not explain all aspects of pattern formation. Therefore, continuous refinement of the model is necessary. For example, the effect of the top wall is not considered in our model. Locations of cell wall remodeling are likely correlated with the type of adjacent cytoskeleton, but we do not have direct experimental evidence to support this. In addition, the molecular nature of the signaling molecule remains to be elucidated. Auxin is a good candidate, but there are some inconsistent observations between its properties and the predictions of our model. For example, our interface equation model assumed that signaling molecule acted at a very short range (approximately 1 μm). Auxin is, however, regarded as a long-range signal. Imaging of morphogen diffusion dynamics during development has recently become possible in animal models [36,37]. The observation of auxin diffusion dynamics is necessary to experimentally verify the interface equation model.

## Supporting Information

S1 Fig Explanation for the G-type Fourier descriptor and ratio of the wavenumber shown in Fig 2f. (TIFF)

S2 Fig Explanation for the scatter diagram shown in Fig 5b and S3 Fig. (TIFF)

S3 Fig Relationship between cell size and the angles of a three-way junction in samples 2 and 4 days after sowing. Large, more developed cells were more likely to have angles close to 120°. Pixel size = 0.49 μm^2^; RMSD, root-mean-square deviation.
(TIFF)

S4 Fig Statistical analysis of three-way junction angle during development. After sowing at 2, 4 and 7 days, cell area and three-way junction angle RMSD of all epidermal cells and all guard cells were analyzed (see Fig 3b and S3 Fig). Cell population was divided in half by cell area. At 4 and 7 days, three-way junctions of large cells approach 120°. Comparison between large cells at 2 and 4 days also suggests a trend of approaching 120° through leaf development. Comparison between large cells at 4 and 7 days also revealed a similar trend. RMSD, root-mean-square deviation.
(TIFF)

S1 Code *Mathematica* source code for the cell wall pattern formation. The code includes numerical simulation of the model described in the main text, and visualization of the ROP activity.
(NB) Click here for additional data file.
