# Supplementary figures and images for: A Theoretical Model of Jigsaw-Puzzle Pattern Formation by Plant Leaf Epidermal Cells
Source: PLoS Comput Biol. 2016 Apr 7;12(4):e1004833. doi: 10.1371/journal.pcbi.1004833 (PMC4824374; doi:10.1371/journal.pcbi.1004833)

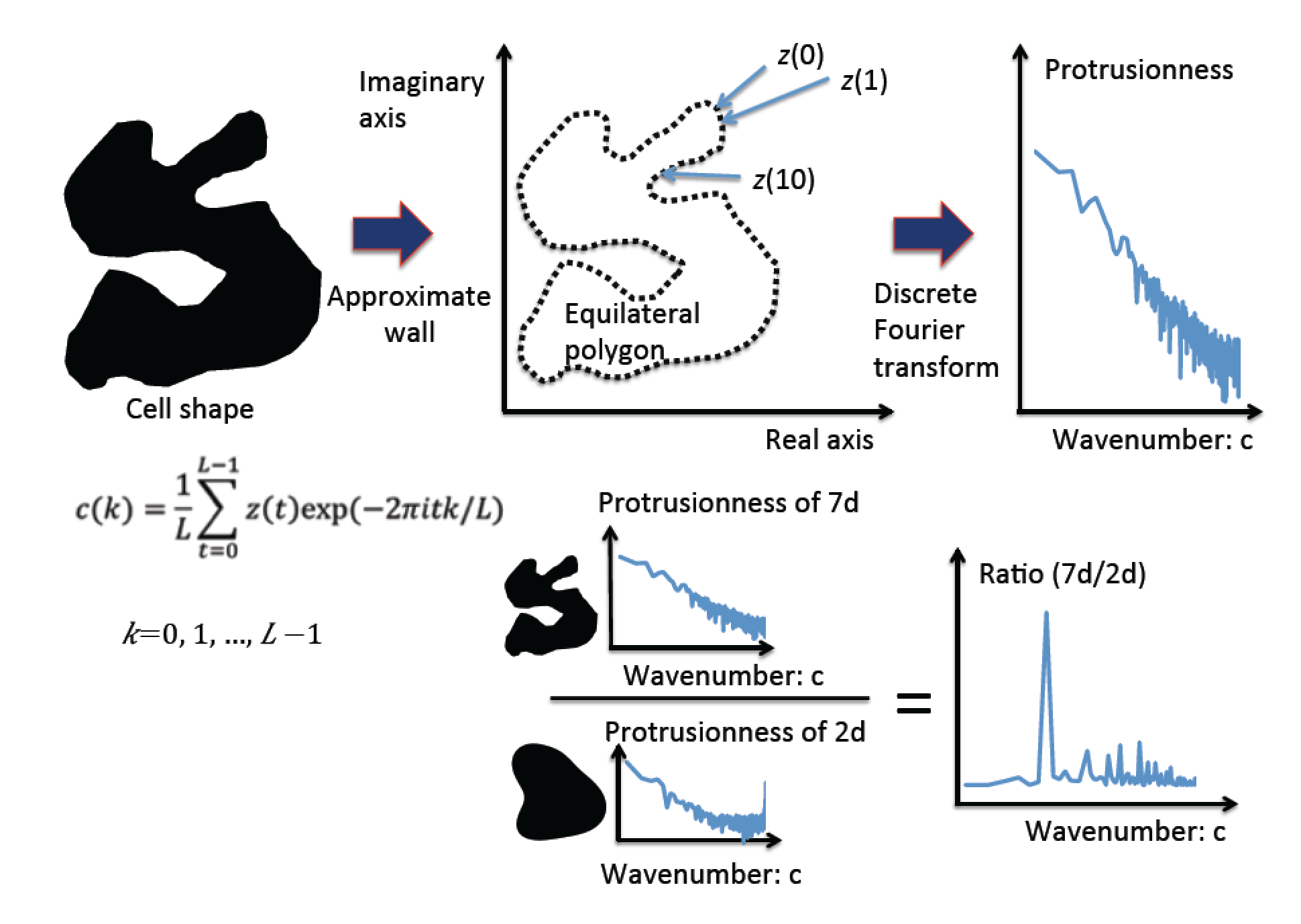

Supplement: S1 Fig — (TIFF) [file pcbi.1004833.s001.tiff]

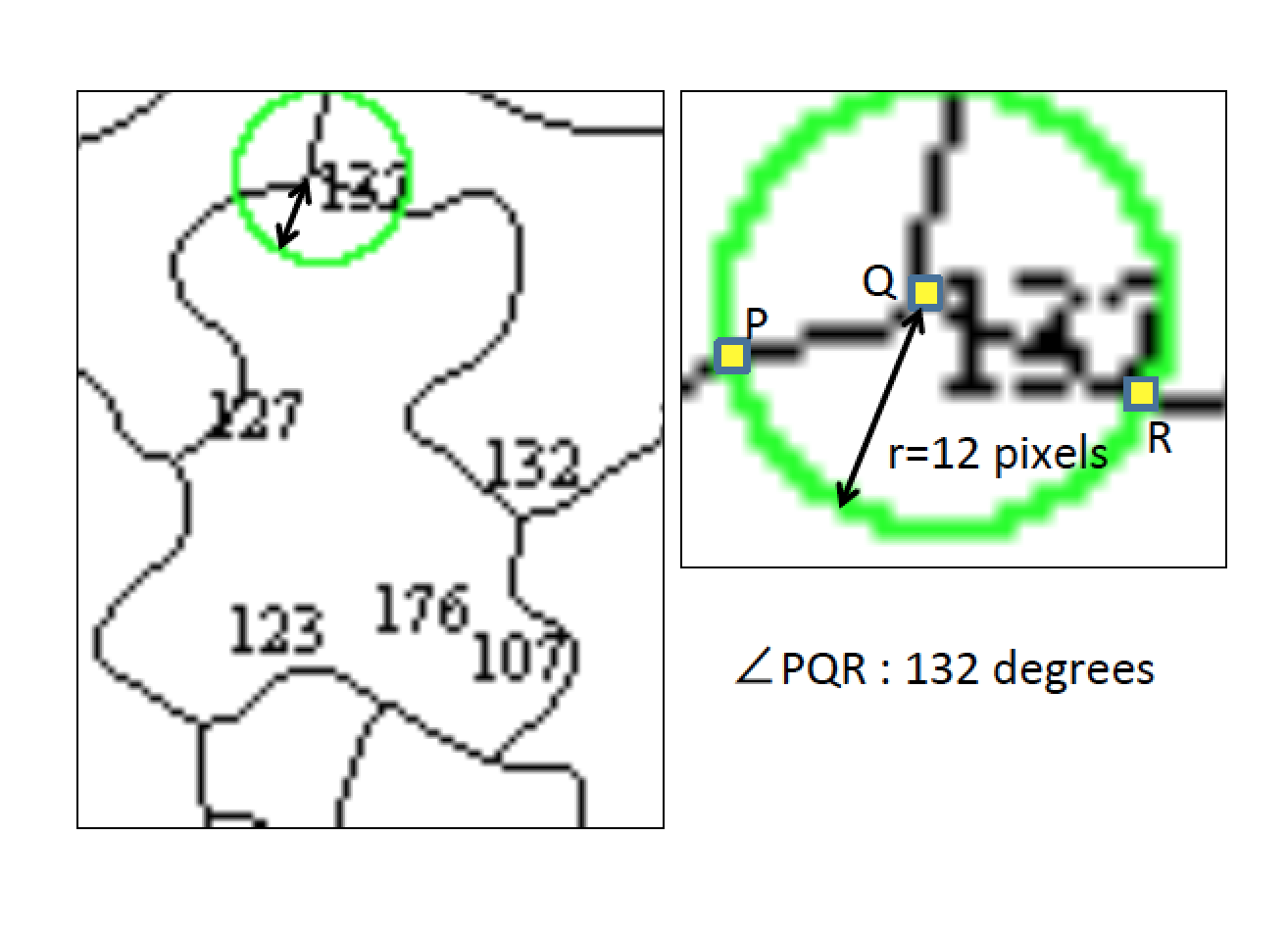

Supplement: S2 Fig — (TIFF) [file pcbi.1004833.s002.tiff]

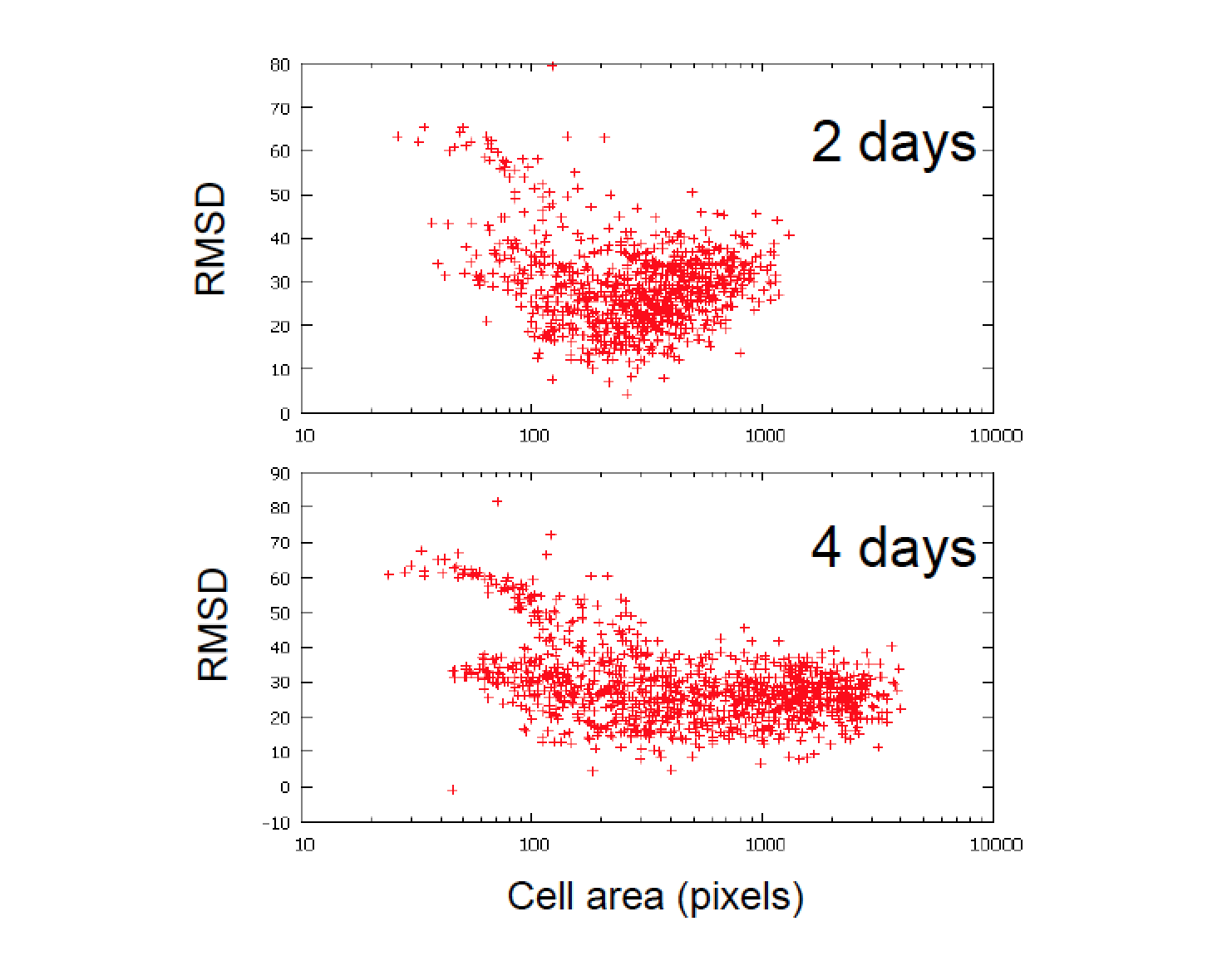

Supplement: S3 Fig — Large, more developed cells were more likely to have angles close to 120°. Pixel size = 0.49 μm2; RMSD, root-mean-square deviation. (TIFF) [file pcbi.1004833.s003.tiff]

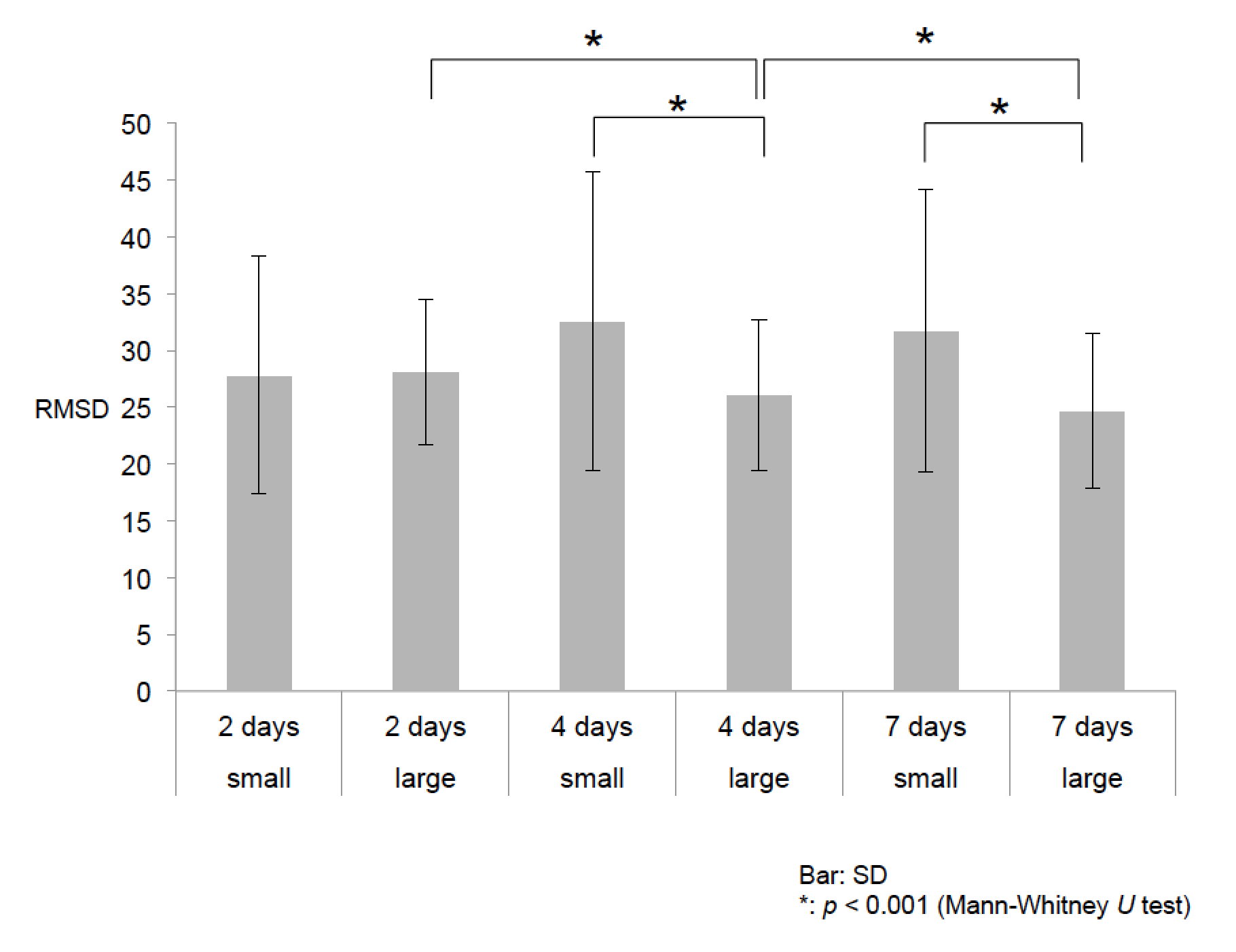

Supplement: S4 Fig — After sowing at 2, 4 and 7 days, cell area and three-way junction angle RMSD of all epidermal cells and all guard cells were analyzed (see Fig 3b and S3 Fig). Cell population was divided in half by cell area. At 4 and 7 days, three-way junctions of large cells approach 120°. Comparison between large cells at 2 and 4 days also suggests a trend of approaching 120° through leaf development. Comparison between large cells at 4 and 7 days also revealed a similar trend. RMSD, root-mean-square deviation. (TIFF) [file pcbi.1004833.s004.tiff]
